# Neonatal Sepsis: Etiology, Antimicrobial Susceptibility, and Treatment Outcomes in a Tertiary Hospital in Jos, Nigeria

**DOI:** 10.4269/ajtmh.24-0127

**Published:** 2025-11-20

**Authors:** David Danjuma Shwe, Udochukwu Michael Diala, Patience Ungut Kanhu, Henry Habila, Olushola Emily Jeremiah, Fatima Joy Baba, Ruth Adah, Bose O. Toma, Stephen Oguche, Tina M. Slusher, Beth K. Thielen, Anne M. White

**Affiliations:** ^1^ Department of Paediatrics, Faculty of Clinical Sciences, College of Health Sciences, University of Jos, Jos, Nigeria;; ^2^ Department of Paediatrics, Jos University Teaching Hospital, Jos, Nigeria;; ^3^ Department of Paediatrics, Modibbo Adama University Teaching Hospital, Yola, Nigeria;; ^4^ Department of Pediatrics, Division of Critical Care, Global Pediatrics Program, University of Minnesota, Minneapolis, Minnesota;; ^5^ Department of Pediatrics, Division of Critical Care, Hennepin Healthcare, Minneapolis, Minnesota;; ^6^ Department of Pediatrics, Division of Infectious Diseases, University of Minnesota, Minneapolis, Minnesota;; ^7^ Department of Pediatrics, Division of Neonatology, University of Minnesota, Minneapolis, Minnesota

## Abstract

Sepsis is a leading cause of neonatal mortality. Current knowledge of etiology, antimicrobial susceptibility, and outcomes provides evidence for judicious antimicrobial use. The aim for the present study was to identify etiologic organisms, antimicrobial susceptibility, and treatment outcomes at a tertiary hospital in Jos, Nigeria. A retrospective case review of neonates hospitalized for sepsis was conducted between August 25, 2017 and December 31, 2020. Clinical and laboratory data were collected from 1,984 neonates admitted, of whom 516 (26%) were diagnosed with neonatal sepsis (NNS). The clinical and blood culture data were available for 380 (74%) neonates, of whom 226 (60%) were male. The majority (63%) were diagnosed with early**-**onset sepsis, of whom 146 (38%) had severe sepsis. The mean age of the mothers was 29.5 ± 5.5 years. Of the 207 cultures obtained, 87 (43%) yielded pure isolates, with 50 (58%) of these being Gram**-**positive. For neonates born outside the study hospital, 6/36 (17%) were methicillin**-**sensitive, compared with 6/44 (14%) neonates born at the study hospital. Gram**-**negative isolates, predominantly *Klebsiella pneumoniae*, grew in 36 (41%) of all positive cultures (27/87; 31%). More organisms were sensitive to piperacillin–tazobactam (19/19; 100%), gentamicin (21/27; 78%), imipenem (4/8; 50%), and ceftazidime (17/28; 61%) than to the other antibiotics tested. Mortality in all patients with proven or presumed NNS was 31/380 (8%), with increased mortality in those without cultures (8/71; 11% versus 39/71; 55%). Neonatal sepsis-related mortality is high in the study center in Jos, Nigeria. Additional work is needed to mitigate NNS mortality and the rising problem of antimicrobial resistance.

## INTRODUCTION

Neonatal sepsis (NNS) is one of the leading causes of neonatal mortality globally, accounting for nearly 780,000 of the 3 million annual deaths.^1^ Survivors of severe NNS have an increased risk of compromised quality of life due to cerebral palsy and neurodevelopmental delays.^2^ Compared with high-income countries, resource-limited countries disproportionately bear the greatest burden of NNS-related morbidity and mortality,^3^ suggesting disparity in access to preventive, diagnostic, and therapeutic services for these neonates. The incidence of NNS in Nigeria remains at 39 per 1,000 live births, ranking among the highest in the world.^4^

Neonatal sepsis is classified as early-onset or late-onset. Early-onset sepsis occurs within 72 hours of birth, usually resulting from pathogens acquired maternally following chorioamnionitis, peripartum pyrexia, or untreated prolonged rupture of fetal membranes. Conversely, late-onset sepsis occurs at least 72 hours after birth, with etiology largely due to nosocomial infections, including those associated with indwelling catheters and community-acquired pathogens.^5^^,^^6^ The etiology of NNS differs substantially across geographic areas, as reported by Downie et al. and Okomo et al. in a systematic review and meta-analysis of studies from 26 countries across sub-Saharan Africa involving more than 80,000 neonates. The results revealed a predominance of *Staphylococcus aureus* (*S. aureus*), *Klebsiella* spp., and *Escherichia coli* (*E. coli*), unlike in high-income countries, where group B *Streptococcus* was the predominant organism.^7^^,^^8^

Multidrug-resistant (MDR) pathogens, including methicillin-resistant *S. aureus* (MRSA), have long been identified as a public health threat to newborn health and survival.^9^ The diagnosis and management of NNS is challenging in many resource-limited settings because of the lack of laboratory capacity to perform specific, accurate, and timely laboratory testing, including cultures, to guide therapy.^1^^–^^3^

In a 2004 study of 122 neonates with clinical suspicion of sepsis in Jos, north-central Nigeria, 66 (54.1%) positive culture results were obtained; *S. aureus* was the predominant Gram-positive isolate in 42 (36.8%) cultures, and 72 (63.2%) cultures resulted in the growth of Gram-negative isolates, predominantly *E. coli*. Most isolates in the 2004 study were sensitive to gentamicin and resistant to cephalosporins.^9^ In 2015, a similar study conducted in Jos revealed a lower rate of positive cultures (34.4%).^10^ In the 2015 study, Gram-positive isolates, predominantly *S. aureus*, made up 41.3% of cases, whereas *Klebsiella pneumoniae* (*K. pneumoniae*) was identified in 58.7% of cases. The same study revealed MDR coagulase-negative *Staphylococcus* culture rates of 26.1%, and MRSA was identified in an additional 60% of cases.^10^ The clinical outcomes of NNS were not examined in relation to the observed bacterial phenotypes in either the 2004 or 2015 study.

Current data on culture-proven NNS, antimicrobial susceptibility, and NNS treatment outcomes among hospitalized neonates in the study area are significantly lacking. These data are required to inform evidence-based treatment decisions. In the present study, it was hypothesized that etiologic isolates of NNS and their antimicrobial resistance profile in Jos, Nigeria, would not differ substantially from previously reported phenotypes.

## MATERIALS AND METHODS

### Design and site of the study.

The present single hospital-based study included a retrospective review of data collected over 40 months (August 25, 2017 to December 31, 2020) and involved newborns who presented and were hospitalized for NNS at the neonatology unit of Jos University Teaching Hospital (JUTH) in Jos, Nigeria. Jos University Teaching Hospital is a 600-bed tertiary hospital in Plateau State, north-central Nigeria, that serves as a major regional referral center. It receives referrals from the neighboring states of Bauchi, Nasarawa, Taraba, Benue, and Southern Kaduna, which together have a combined population of more than 13 million people.^11^ The JUTH neonatal unit has a 30-bed capacity and manages neonates born at JUTH and those born outside the hospital (e.g., at home, a health clinic, another hospital, or another site).

### Study population.

The current study included all neonates aged 0–28 days who were admitted to the neonatal unit for NNS. Neonates who received antibiotics before admission were excluded.

### Microbiological sample collection.

As is standard practice, a minimum of 1–3 mL of blood was obtained from each study participant for culture from a peripheral vein under aseptic precautions; after collection, the sample was shaken and then immediately transported to the laboratory. Samples were registered and processed on arrival at the laboratory. Blood samples were incubated in a blood culture machine (BACTEC-9050, Becton, Dickinson and Company, Franklin Lakes, NJ). This semiautomatic system flags red and emits a sound when growth is detected in blood cultures. Positive cultures were subsequently subcultured.

Enriched media for subculture included sheep blood, MacConkey, and chocolate agar plates. Media were inoculated, incubated at 37°C, and examined for growth after 24–48 hours. Isolates were identified via Gram stain, colony morphology, and biochemical properties, including catalase, DNAse agar, mannitol salt agar, and hemolysis, as appropriate. Triple sugar iron, lysine iron agar, motility indole, ornithine, citrate, urease, and oxidase were used for Gram-negative bacilli.^12^ All media and biochemical reactions were quality-assured and -controlled according to American Type Culture Collection standards.^13^

Antibiotic susceptibility tests were performed using the Kirby–Bauer disk diffusion method, as specified by the Clinical and Laboratory Standards Institute.^14^ The inhibition zones were measured and interpreted in accordance with the Clinical and Laboratory Standards Institute’s recommendations.^15^ Multidrug-resistant bacteria were defined as those resistant to three or more antimicrobial classes.^12^ A fixed number of antimicrobials were tested against each isolate identified, according to local laboratory protocol and based on availability.

### Treatment of neonates with sepsis.

Neonates born at JUTH and admitted for NNS initially received ampicillin and ceftazidime combination therapy. Neonates born outside of JUTH initially received ampicillin and gentamicin combination therapy. Antibiotics were continued while awaiting the return of blood culture results from the hospital’s main laboratory. The turnaround time for blood culture was 2–7 days. The first-line antimicrobials could be changed to second-line antimicrobials (e.g., ciprofloxacin plus gentamicin) in the absence of clinical evidence of improvement despite adequate therapeutic doses of the initial antibiotics; these decisions were guided by a previous unit antibiogram and United Nations Children’s Fund Recommendations on Sepsis Diagnostics.^14^

### Study outcomes.

The primary outcome was the number of culture-proven cases of NNS. The secondary outcomes included the identification of etiologic agents of NNS, antimicrobial susceptibility profiles of the isolates, and NNS treatment outcomes.

### Definition of key terms.

Neonatal sepsis was diagnosed by treating physicians on the basis of clinical and laboratory diagnostic criteria, including a history of either fever (≥37.5°C) or hypothermia (≤35.5°C), fast breathing (≥60 breaths per minute), not feeding well, severe chest in-drawing, movement only when stimulated, convulsion, lethargy, or unconsciousness, a history of maternal risk factors, and laboratory studies such as blood culture, total leukocyte count (<5.0 × 10^9^ cells/L or >20.0 × 10^9^ cells/L), absolute neutrophil count (<2.0 × 10^9^ cells/L), platelet count (<150 × 10^9^ cells/L), and cerebrospinal fluid analysis.^16^

Gestational age was determined using the mother’s last menstrual period, an early ultrasound scan, or a modified Ballard score. Prematurity was defined as a gestational age of <37 completed weeks. Term was defined as a gestational age of 37 to 42 completed weeks, and post-term was defined as a gestational age of >42 completed weeks.

Severe NNS (presumed) was defined on the basis of the WHO’s integrated management of childhood illness severity indicator, which is characterized by the presence of one or more of the following symptoms: abnormal temperature (>38.0°C or <35.5°C), hypoxemia (oxygen saturation of <90% in room air while awake), severe anemia (as defined by age), grunting, severe respiratory distress (respiratory rate of <40 breaths/minute or >60 breaths/minute), leukocytosis/leukopenia (as defined by age), cyanosis, convulsions, jaundice, delayed capillary refill, coma, inability to feed, oliguria (urine output of <0.5 mL/kg/hour).^17^^,^^18^

Culture-proven bloodstream infection (sepsis) was defined as a positive blood culture result in the presence of clinical signs and symptoms of infection. Probable sepsis was defined as the presence of clinical signs and symptoms, along with at least two abnormal laboratory results with negative blood culture results. Possible sepsis was defined as the presence of clinical signs and symptoms of infection, accompanied by increased C-reactive protein or interleukin (IL)-6/IL-8 levels, in the absence of a positive blood culture result. Sepsis was not diagnosed without clinical signs and symptoms of infection or abnormal laboratory results.^19^ Presumed sepsis is the working diagnosis for a neonate who presents with features consistent with NNS before a confirmed diagnosis.

### Data abstraction.

Data abstraction was performed by three co-investigators and two trained research assistants. These research assistants were trained by the lead investigator. For mothers of inborn neonates, any missing maternal records of interest from the unit admission register were retrieved from the mother’s clinical document to update the data collection tool. On average, 30–45 medical records for mothers of inborn neonates were retrieved each week. Any mother–neonate dyad with missing medical records that were deemed significant was excluded. To ensure accuracy and completeness, abstracted data were double-checked by the investigators.

### Data collection tool.

A semi-structured proforma (Supplemental Information) was adapted on the basis of the reviewed literature related to NNS and its known risk factors.^20^ The data collection tool consisted of two sections: Section A, which included biodata, obstetric characteristics, neonatal characteristics, and anthropometric measures, and Section B, which included maternal and family characteristics. Neonatal characteristics included neonatal age and gestational age at admission, sex, birthweight (kg), at-birth vaccination status, admitting diagnosis, known risk factors for sepsis, temperature, admission random blood glucose, sepsis screen results, and duration of hospitalization. Maternal and family characteristics included maternal age (years), highest educational attainment, parity, mode of delivery, place of delivery (inborn or out-born), antenatal clinic attendance, hypertensive and bleeding disorders of pregnancy, HIV, Hepatitis B virus, and Hepatitis C virus status, urinary tract infection or sexually-transmitted infection in pregnancy, prolonged rupture of membranes, chorioamnionitis, history of previous baby (or babies) with omphalitis or NNS, and history of deceased neonates.

### Data processing.

Medical records were serialized by date and coded as culture-positive, culture-negative, or culture not performed. They were double-checked for accuracy and completeness. The data were entered into an Excel spreadsheet (Microsoft Corp., Redmond, WA) and then exported to SPSS version 23.0 (1989, 2015; IBM Corp., Armonk, NY) for analysis. Medical records were reviewed between August 17, 2017 and December 31, 2020, and data extraction was completed within 6 weeks after the end of the review period.

## STATISTICAL ANALYSES

Sociodemographic, obstetric, and perinatal characteristics of the neonates and their mothers were expressed as frequencies and percentages. The mean ± SD was presented as a summary index for neonates’ and their mothers’ ages when data were normally distributed; median and interquartile range were used for data that did not meet the assumptions of normality. Qualitative variables were described using frequencies and proportions and then presented in tables. Pearson’s χ^2^ test was used to test associations between categorical variables. All tests of significance were two-tailed. A significance level of *P* <0.05 indicated a statistically significant difference.

## RESULTS

Of a total of 516 hospitalizations for NNS, data were available for 380 (74%), which were included in the analysis. A total of 77 (15%) were excluded because of incomplete maternal data, and 59 (11%) were excluded because of incomplete clinical and laboratory data. The mean age of mothers was 29.5 ± 5.5 years, with the majority (283/380; 75%) being aged 20–34 years. Most neonates (241/380, 63%) were admitted within 72 hours of life with early onset NNS, and 226/380 (60%) were male (Table 1).

**Table 1:** Demographic characteristics of mothers and neonates

Variable	Frequency (*N* = 380)	Percentage
Age group of mother (year)		
Less than 20	14	3.7
20–34	283	74.5
≥35	83	21.8
Mean age	29.5 ± 5.5	–
ANC attendance		
No	25	6.6
Yes	355	93.4
Parity		
P1	123	32.4
P2-4	206	54.2
Greater than P4	51	13.4
Highest educational attainment		
No formal education	32	8.4
Primary	54	14.2
Secondary	143	37.6
Tertiary	151	39.7
Place of birth		
Inborn*	172	45.3
Out-born	208	54.7
Sex		
Male	226	59.5
Female	154	40.5
Male-to-female ratio	1.5:1	–
Time to NNS onset		
Early	241	63.4
Late	139	36.6
Gestational age at birth (completed weeks)^†^		
Less than 28	21	5.5
28–32	62	16.3
33–37	72	19.0
38–42	216	56.8
Greater than 42	9	2.4
Weight at admission (kg)		
Less than 2.5	157	41.3
2.5–3.99	193	50.8
Greater than 3.99	30	7.9
Temp. at admission (°C)		
Less than 36.5	71	18.7
36.5–37.4	184	48.4
Greater than 37.4	125	32.9
RBS at admission		
Normoglycemia	289	76.1
Hyperglycemia	15	3.9
Hypoglycemia	76	20.0
NNS Severity		
Severe	146	38.4
Not severe	234	61.6
Vaccination status at admission^‡^		
Vaccinated	132	34.7
Unvaccinated	248	65.3

ANC = antenatal clinic; NNS = neonatal sepsis; P = parity; RBS = random blood sugar; Temp = temperature.

* Inborn: hospital birth and never left hospital.

^†^ Preterm: GA less than 37 completed weeks.

^‡^ Vaccinations at birth include Bacillus-Calmette Guerin, oral polio vaccine, and Hepatitis B virus.

The study team monitored the enrolled neonates for 14 sepsis symptom severity indicators, as outlined in the WHO Integrated Management of Childhood Illness guide.^18^ Abnormal axillary temperature (>38.0°C or <35.5°C) was observed in the majority (238/380; 32%) of neonates diagnosed with severe sepsis at admission. In addition, laboratory evidence of acute kidney injury (oliguria) was documented in 11/380 (3%) of the studied neonates (Table 2).

**Table 2:** Neonatal sepsis severity symptoms based on WHO integrated management of childhood illnesses criteria

	Symptoms		
Severity Indicator	Frequency (*N* = 380)	Percentage (%)	Cumulative Percentage (%)
Abnormal temperature (>38.0°C or <35.5°C)	238	32.4	32.4
Hypoxemia (SpO_2_ <90%)	104	14.2	46.6
Severe anemia	61	8.3	54.9
Grunting	48	6.5	61.4
Severe respiratory distress (RR <40/minute or >60/minute)	48	6.5	67.9
Leukocytosis/leukopenia	41	5.6	73.5
Cyanosis	40	5.5	79.0
Convulsions	38	5.2	84.2
Jaundice	37	5.0	89.2
Delayed capillary refill	29	3.9	93.1
Coma	28	3.8	96.9
Inability to feed	12	1.6	98.5
Oliguria: urine output <0.5 mL/kg/hour	11	1.5	100
Total	735	100	100

RR = respiratory rate; SpO_2_ = peripheral capillary oxygen saturation.

### Sepsis etiology.

Of a total of 380 neonates whose records were available for analysis, blood culture specimens were collected and processed for 207 (55%), of which 87 (42%) yielded positive results. Blood cultures were not performed on 173 neonates for various reasons, including the inability of parents to pay for the culture, unavailable blood culture bottles, early deaths before the procedure could be completed, or the physician’s failure to order a blood culture. Antibiotic sensitivity data were not available for 12 cases with positive culture results. Of the 87 culture-proven sepsis cases, 50 (58%) were Gram-positive; among these, the predominant organism was *S. aureus* (28/44; 64%) for neonates born at JUTH and 19/36 (53%) for those born outside of JUTH. Of all *S. aureus* isolates, 6/28 (21%) and 6/19 (32%) were due to MRSA for neonates born at JUTH and outside of JUTH, respectively. A total of 36/87 (41%) Gram-negative isolates were identified, with *K. pneumoniae* being the predominant isolate (27/87; 31%). *Streptococcus pneumoniae* was the least common etiologic agent isolated (1/87, 1%) and was found in one neonate born at JUTH (Table 3). A total of 120 of 207 blood cultures that were sent (58%) yielded negative results in neonates with clinical signs consistent with probable sepsis.

**Table 3:** Etiology and bacteriologic profile for neonatal sepsis

Etiologic Agents	Frequency	*n* = 87/207
	Inborn	Out-born
*Staphylococcus aureus*		
MSSA	6	6
MRSA	6	7
NOS*	16	7
*Klebsiella pneumoniae* ^†^	13	10
*Citrobacter* spp.	2	2
*Escherichia coli*	0 (0.0)	3
*Enterbacter* spp.	0 (0.0)	2
*Streptococcus pneumoniae*	1	0 (0.0)
Total	44 (100)	36 (100)

MRSA = methicillin-resistant *Staphylococcus aureus*; MSSA = methicillian-sensitive *Staphylococcus aureus*; NOS = not otherwise specified.

* Three missing data points for inborn and out-born.

^†^ Four missing data points for inborn and out-born.

### Sensitivities.

The 12 methicillian-sensitive *S. aureus* (MSSA) isolates detected exhibited the greatest sensitivity (>80%) to gentamicin, ceftriaxone, cefixime, cefuroxime, piperacillin–tazobactam, amoxicillin–clavulanic acid, and imipenem. The 12 MRSA isolates detected exhibited the greatest sensitivity (>80%) to gentamicin and chloramphenicol. The single positive *Streptococcus* isolate exhibited 100% sensitivity to piperacillin–tazobactam and amoxicillin–clavulanic acid (Tables 4–6).

**Table 4:** Antimicrobial susceptibility of organisms for inborn neonates

Isolated Organism	Isolates	Gentamicin	Piperacillin–tazobactam	Ceftazidime	Amoxicillin–clavulanic acid	Azithromycin	Ciprofloxacin	Imipenem	Chloramphenicol	Ampicillin-cloxacillin	Cefixime	Penicillin	Cefuroxime	Levofloxacin	Oxacillin	Erythromycin	Ceftriaxone	Cotrimoxazole
Gram-positive organisms																		
MRSA	6	2/3	–	0/3	–	1/5	2/4	–	3/3	–	–	0/1	–	–	0/2	–	0/1	–
MSSA	6	3/3	2/2	–	1/1	2/2	0/4	–	1/3	–	2/2	0/5	1/1	–	–	–	–	–
*S. aureus* NOS	16	–	–	–	–	9/9	3/7	–	4/8	–	–	1/6	–	–	–	–	–	–
*Streptococcus* spp.	1	–	1/1	–	0/1	–	–	–	–	–	–	–	–	–	–	–	–	–
Gram-negative organisms																		
*Citrobacter* spp.	2	2/2	2/2	–	0/2	–	–	–	–	–	0/2	–	–	–	–	–	0/2	–
*Klebsiella* spp.	13	2/3	4/4	4/6	0/8	0/3	1/7	0/2	0/4	–	–	0/2	0/3	1/1	–	–	0/4	–

MRSA = methicillin-resistant *Staphylococcus aureus*; MSSA = methicillian-sensitive *Staphylococcus aureus*; NOS = not otherwise specified; *S. aureus* = *Staphylococcus aureus*.

**Table 5:** Antimicrobial susceptibility of organisms for out-born neonates

Isolated Organism	Isolates	Gentamicin	Piperacillin–tazobactam	Ceftazidime	Amoxicillin–clavulanic acid	Azithromycin	Ciprofloxacin	Imipenem	Chloramphenicol	Ampicillin–cloxacillin	Cefixime	Penicillin	Cefuroxime	Levofloxacin	Oxacillin	Erythromycin	Ceftriaxone	Cotrimoxazole
Gram-positive organisms																		
MRSA	6	4/4	–	0/2	0/2	2/6	4/4	–	2/2	–	–	–	0/2	–	–	–	–	–
MSSA	6	4/6	2/2	1/1	1/1	2/6	2/4	2/2	1/1	–	–	0/1	2/2	–	–	0/1	1/1	–
*S. aureus* NOS	7	1/1	2/2	2/2	1/2	1/1	0/1	–	–	–	–	–	0/2	–	–	–	–	–
Gram-negative organisms																		
*E. coli*	3	1/1	–	3/3	1/2	–	3/3	–	–	–	1/1	–	0/1	1/1	–	–	0/1	–
*Citrobacter* spp.	2	–	2/2	0/2	–	–	0/2	0/2	–	–	0/2	–	–	–	–	–	–	–
*Klebsiella* spp.	10	0/2	2/2	3/3	0/5	0/2	2/4	—	–	0/2	1/1	–	0/2	1/3	–	–	0/2	–
*Enterobacter* spp.	2	2/2	2/2	0/2	2/2	–	0/2	2/2	–	–	–	–	–	–	–	–	–	–

*E. coli* = *Escherichia coli*; MRSA = methicillin-resistant *Staphylococcus aureus*; MSSA = methicillian-sensitive *Staphylococcus aureus*; NOS = not otherwise specified; *S. aureus* = *Staphylococcus aureus*.

**Table 6:** Antimicrobial susceptibility of organisms for neonates not documented inborn or out-born

Isolated Organism	Isolates	Gentamicin	Piperacillin–tazobactam	Ceftazidime	Amoxicillin–clavulanic acid	Azithromycin	Ciprofloxacin	Imipenem	Chloramphenicol	Ampicillin–cloxacillin	Cefixime	Penicillin	Cefuroxime	Levofloxacin	Oxacillin	Erythromycin	Ceftriaxone	Cotrimoxazole
Gram-positive organisms																		
*S. aureus* NOS	3	–	–	–	–	0/1	0/3	–	0/3	–	–	0/3	–	–	–	–	–	–
Gram-negative organisms																		
*Klebsiella* spp.	4	–	–	4/4	0/4	0/4	0/4	–	0/2	–	–	0/2	–	–	0/2	–	–	–

NOS = not otherwise specified; *S. aureus* = *Staphylococcus aureus*.

The *Klebsiella* spp. isolates exhibited sensitivity (>80%) to ceftazidime and piperacillin–tazobactam. A similar pattern of reduced antimicrobial sensitivity was observed in *Citrobacter* spp., which were sensitive only to gentamicin and piperacillin–tazobactam. *Escherichia coli* isolates exhibited >80% sensitivity to gentamicin, ceftazidime, cefixime, levofloxacin, and ciprofloxacin. The two *Enterobacter* spp. isolates exhibited >80% sensitivity to gentamicin, piperacillin–tazobactam, amoxicillin–clavulanic acid, and imipenem (Tables 4–6).

### Risk factors for neonatal mortality.

Of a total of 71 cases that resulted in mortality, eight (11%) had culture-proven sepsis. A statistically significant negative correlation between culture-proven sepsis and mortality events was observed (regression coefficient = –0.137; *P* = 0.007; Table 7). In addition, the availability of culture-proven sepsis test results significantly reduced the likelihood of sepsis mortality (χ^2^ = 4.101; degree of freedom = 1; *P* = 0.043).

**Table 7:** Displays the distribution of mortality by blood culture tests and place of birth

Variable		Inborn Mortality	Out-born Mortality	Total
Blood culture	Positive	3 (9.1)	5 (13.2)	8 (11.3)
	Negative	8 (24.2)	16 (42.1)	24 (33.8)
	Not performed	22 (66.7)	17 (44.7)	39 (54.9)
Total		33 (100)	38 (100)	71 (100)

R = −0.137; *P* = 0.007; χ^2^ = 4.101; df = 1; *P* = 0.043.

## DISCUSSION

Nearly one-third of neonatal admissions at a tertiary referral hospital with a dedicated NICU located in north-central Nigeria were diagnosed with NNS. *Staphylococcus aureus* was the etiologic agent in more than half of culture-proven sepsis cases. Overall, fewer than half of the blood culture tests yielded positive results. The majority of neonates received empirical broad-spectrum antimicrobials without blood culture results because of their families’ inability to pay for laboratory services or supply chain issues resulting in a lack of available blood culture bottles. As in other studies from sub-Saharan Africa, a substantial proportion of positive isolates exhibited antibiotic resistance.^7^ The neonatal mortality rate due to proven and suspected sepsis in the present study was unacceptably high. The findings of the current study have significant clinical practice and policy implications for both neonatologists and healthcare managers.

The rate and severity of NNS were high in the present study and in other studies conducted in Nigeria, including those from Abeokuta, Gwagwalada, and Gusau, making this an important public health concern.^20^^–^^22^ Neonatal sepsis is the third leading cause of neonatal mortality in low- and middle-income countries (LMICs) like Nigeria, where infrastructure for disease surveillance and diagnostics is significantly lacking.^1^ Compared with older children, neonates, especially preterm ones, are immune-naïve, making them more susceptible to infections.^23^

Although the etiologic agents of NNS identified in the present study were diverse, several organisms were established as the most commonly found. In more than half of culture-proven cases, Gram-positive bacteria were predominant, with *S. aureus* being the most common. Almost 25% of the isolates were either MSSA or MRSA, whereas 50% were *S. aureus* not otherwise specified. In developed countries, both MSSA and MRSA have caused outbreaks in neonatal intensive care settings, indicating the need for rigorous infection prevention and other approaches to reduce the risk of hospital-acquired infections with this organism.^24^^–^^26^ *Enterobacter* spp. have long been recognized as having an ampC gene that confers inducible resistance to commonly used, safe, and affordable therapeutic agents such as penicillin and cephalosporins.^10^^,^^27^ *Klebsiella* spp., a Gram-negative organism, was the predominant isolate among nearly one-third of the etiologic agents of sepsis identified in the present study.

In this study setting, as in most LMIC settings,^28^ it is standard of care to commence antibiotics for neonates with suspected severe NNS, guided by previously established bacteriologic profiles. This clinical practice is increasingly problematic in the face of drastically increasing antibiotic resistance. In addition, studies have clearly revealed that inappropriate antibiotic use increases the risk of the emergence and spread of antimicrobial resistance, colonization by resistant pathogens, necrotizing enterocolitis, wheezing, and obesity in later childhood.^27^^–^^32^ Active surveillance and metagenomics studies are urgently needed to better understand the intraspecies diversity of MDR phenotypes and reduce neonatal morbidity and mortality, the duration of hospitalization, and the duration of antibiotic treatment. Reversing the current trend of MDR progression is necessary to prolong the therapeutic usefulness of available, reasonably priced antimicrobials and decrease the development of further resistance.

The diagnosis and management of NNS is complex and challenging in resource-limited settings like Jos, Nigeria. This is particularly true given the nonspecific symptoms and signs of NNS, coupled with universal guidelines for therapy in the absence of adequate laboratory infrastructure for accurate and time-sensitive diagnostics. Frequent inability to perform blood cultures because of parents’ lack of funds or the unavailability of blood culture bottles results in fewer blood cultures being performed.^33^ Even when blood cultures are performed, interpretation of these findings is limited by incomplete sensitivity data, which is at least in part related to frequent stock-outs of critical laboratory reagents and consumables, including antibiotic-impregnated disks. Previous work from Kenya revealed that treatment decisions for only 0.1% of patients prescribed antimicrobials were based on antimicrobial susceptibility testing.^34^ Improving access to blood culture in LMICs requires pragmatic thinking, and manufacturers have a responsibility to consider LMIC contexts and improve accessibility, such as by extending shelf lives and testing performance under different climatic conditions.^35^^–^^37^ These constraints are reflected in the high proportion of neonates diagnosed with “probable” or “presumed” sepsis and the lack of blood culture results for the majority of neonates in the present study. Point-of-care diagnostics for sensitive sepsis biomarkers, such as C-reactive protein, procalcitonin, IL-6, IL-8, and, more recently, next-generation sequencing-based pathogen detection, could potentially serve as reliable alternatives to blood cultures in neonates; however, further studies are needed.^36^ These biomarkers are routinely used in high-income countries, where they are more widely available and used to help guide NNS treatment.^38^ These findings further highlight the need to expand diagnostic access to healthcare services in LMICs to improve equity in neonatal health and survival.

Mortality events were more than threefold higher among neonates for whom blood cultures were not performed compared with those for whom blood cultures were performed. The availability of culture-proven sepsis tests significantly reduced the likelihood of NNS-mortality events. This finding could be related to the ability to tailor antimicrobial treatment to the specific organism responsible in cases with a known bacterial etiology of sepsis. Other explanations include that families unable to pay for blood cultures may live in greater poverty than those able to afford them, contributing to worse overall health among neonates and an increased overall risk of mortality. Finding creative and sustainable ways to fund blood cultures and laboratory testing, including biomarker identification, even when families cannot pay, is likely to reduce neonatal mortality. In addition, it may reduce costs through targeted and appropriate medication therapy and decreased hospital length of stay.

Compared with MSSA, fewer effective therapeutic options were available for MRSA, as expected. Limited therapeutic options were also available for *Klebsiella* spp., *Citrobacter* spp., and *Enterobacter* spp. Proven infection prevention interventions, such as optimal adherence to hand hygiene practices by healthcare workers, may reduce nosocomial transmission of infections and halt further spread of resistant pathogens; this is essential for prolonging the therapeutic usefulness of available, safe, and affordable antimicrobials in LMICs.

Compared with previous studies conducted in Jos, the 43% blood culture-positive rate in the present study was lower than 54% reported in 2004 but higher than 34% reported in 2015.^9^^,^^10^ *Staphylococcus aureus* remains the predominant Gram-positive isolate, whereas *K. pneumoniae* and *E. coli* are still the most common Gram-negative bacterial etiologies, as shown in previous studies conducted in the area.^9^^,^^10^ These observations support the earlier hypothesis stating that the etiologic agents for NNS in Jos, Nigeria, are similar to previously identified organisms. In addition, antimicrobial sensitivity remained similar, with gentamicin among the most effective agents in both previous studies and the current study.

Both etiology and antimicrobial susceptibility profiles from the present study support previous reports from Kano, northwest Nigeria, but differ significantly from those of a similar study conducted in south Nigeria, where ofloxacin-resistant *K. pneumoniae* was isolated as the most common bacterial etiology for NNS.^20^^,^^38^ Similar NNS etiologies were reported in South Africa and Uganda, where *Staphylococcus* spp. (one-third of which were MRSA), extended-spectrum beta-lactamase *K. pneumoniae*, *Enterococci* spp., and *E. coli* were the most frequently identified isolates. In addition, the authors of previous studies isolated *Acinetobacter baumannii* and fluconazole-resistant *Candida parapsilosis*, which were not found in the present study.^39^^,^^40^

In Southeast Asia, nearly two-thirds of isolates were Gram-negative pathogens (including *K. pneumoniae* and *E. coli*), whereas 46.5% were MRSA. This report differed significantly from the present study.^41^ The reason for these differences remains unclear.

The observed neonatal mortality rate for culture-proven sepsis in the current study is high, but it is comparable to that of studies conducted in different parts of Nigeria, ranging from 6.6% reported in Gusau, northern Nigeria, to 37.9% reported in northwest Nigeria.^20^^,^^21^^,^^42^^–^^46^ This disparity may be due to disparities in access to specialized newborn diagnostics and preventive care services within Nigeria, as well as differences in place of delivery, cultural practices, and access to newborn–maternal health information. Furthermore, the definition of sepsis and its severity may also play an important role. Additionally, whereas NNS mortality was classified by onset time and cause-specific mortality in the studies cited above, culture-proven sepsis was compared with all-cause mortality in the current study.

As noted above, the present study was limited by the fact that blood cultures were not performed for a significant proportion of neonates treated for sepsis. Additionally, the use of antibiograms was limited to what was available at the microbiology laboratory and was not tailored specifically to neonates. These considerations, as well as decisions regarding empirical antibiotic therapy, can be better assessed in the context of a future prospective study.

Observations from the present study provide critical information for clinicians, policy actors, and healthcare managers regarding the diagnosis and management of NNS in the study setting. For clinicians, therapeutic decisions should be guided by current antimicrobial profiles based on blood culture results. In addition, blood cultures should be requested before antibiotics are started, and the cost should be covered by the hospital to avoid placing a burden on families. For healthcare managers and policy actors, substantial investment should be made in laboratory infrastructure to expand access to diagnostics and limit excess NNS-attributable mortality due to probable and unproven sepsis. Collaboration among international partners and colleagues in LMICs should be focused on creative and sustainable solutions. International corporations must partner with organizations on the ground in LMICs to develop strategies to address challenges related to cost and access to essential diagnostics.

## CONCLUSION

The current study provides evidence to assist local clinicians and healthcare managers in guiding NNS treatment decisions in Jos, Nigeria, in many LMICs, and possibly in high-income countries worldwide. Up-to-date epidemiologic data, such as those provided in the present study, are also helpful in increasing the global body of evidence on the prevalence, etiology, and antimicrobial susceptibility of NNS worldwide. The current study also highlights the need for biomarkers, metagenomic studies, and other novel testing to improve the diagnosis and treatment of sepsis and improve neonatal outcomes.

## Supplemental Materials

10.4269/ajtmh.24-0127 Supplemental Materials

## Data Availability

The data used to support the findings of this study are available from the corresponding author upon request.

## References

[1] Liu L, , 2012. Global, regional, and national causes of child mortality: An updated systematic analysis for 2010 with time trends since 2000. Lancet 379: 2151–2161. 22579125 10.1016/S0140-6736(12)60560-1

[2] Stoll BJ Hansen NI Adams-Chapman I Fanaroff AA Hintz SR Vohr B Higgins RD; National Institute of Child Health and Human Development Neonatal Research Network, 2004. Neurodevelopmental and growth impairment among extremely low-birth-weight infants with neonatal infection. JAMA 292: 2357–2365. PMID: 15547163. 15547163 10.1001/jama.292.19.2357

[3] Stoll BJ, , 2011. Early onset neonatal sepsis: The burden of group B Streptococcal and *E. coli* disease continues. Pediatrics 127: 817–826. 21518717 10.1542/peds.2010-2217 PMC3081183

[4] National Populations Commission [Nigeria] and ICF, 2019. Nigeria Demographic and Health Survey 2018. Abuja, Nigeria, and Rockville, MD: National Populations Commission and ICF.

[5] Puopolo KM Draper D Wi S Newman TB Zupancic J Lieberman E Smith M Escobar GJ, 2011. Estimating the probability of neonatal early-onset infection on the basis of maternal risk factors. Pediatrics 128: e1155–e1163. 22025590 10.1542/peds.2010-3464 PMC3208962

[6] Stoll BJ, , 2002. Lateonset sepsis in birth weight neonates: The experience of the NICHD Neonatal Research Network. Pediatrics 110: 285–291. 12165580 10.1542/peds.110.2.285

[7] Downie L Armiento R Subhi R Kelly J Clifford V Duke T, 2013. Community-acquired neonatal and infant sepsis in developing countries: Efficacy of WHO’s currently recommended antibiotics—Systematic review and meta-analysis. *Arch Dis Child* 98: 146–154. PMID: 23142784 23142784 10.1136/archdischild-2012-302033

[8] Okomo U Akpalu ENK Le Doare K Roca A Cousens S Jarde A Sharland M Kampmann B Lawn JE, 2019. Aetiology of invasive bacterial infection and antimicrobial resistance in neonates in sub-Saharan Africa: A systematic review and meta-analysis in line with the STROBE-NI reporting guidelines. *Lancet Infect Dis* 19: 1219–1234. 31522858 10.1016/S1473-3099(19)30414-1

[9] Bode-Thomas F Ikeh EI Pam SD Ejeliogu EU, 2004. Current aetiology of neonatal sepsis in Jos University Teaching Hospital. Niger J Med 13: 130–135. 15293830

[10] Onyedibe KI Bode-Thomas F Nwadike V Afolaranmi T Okolo MO Uket O Diala UM Da’am CK Egah DZ Banwat EB, 2015. High rates of bacteria isolates of neonatal sepsis with multidrug resistance patterns in Jos, Nigeria. *Ann Pediatr Child Health* 3: 1052.

[11] Nigeria National Population Commission, 2020. National Population Commission. Nigerian Projected Population by State, Sex and Year. Available at: https://www.nigerianstat.gov.ng/pdfuploads/DEMOGRAPHIC%20BULLETIN%202020.pdf. Accessed June 21, 2025.

[12] Magiorakos A-P, , 2012. Multidrug-resistant, extensively drug-resistant and pandrug-resistant bacteria: An international expert proposal for interim standard definitions for acquired resistance. Clin Microbiol Infect 18: 268–281. 21793988 10.1111/j.1469-0691.2011.03570.x

[13] Miller M Holmes HT Krisher K, 2003. Susceptibility test methods: Dilution and disc diffusion methods. Murray PR Pfaller MA Jorgensen JH Yolken RH, eds. Manual of Clinical Microbiology, 8^th^ ed. Washington, DC: ASM Press, 1526–1543.

[14] United Nations Children’ s Fund, 2020. *Sepsis Diagnostics – Infection Prevention and Control, 1^st^ Edition*. Available at: https://www.unicef.org/supply/media/2946/file/sepsis-diagnostic-use-cases.pdf. Accessed October 28, 2025.

[15] Clinical and Laboratory Standards Institute, 2015. Performance Standards for Antimicrobial Susceptibility Testing; Twenty- Fifth Informational Supplement (M100-S25). Wayne, PA: Clinical and Laboratory Standards Institute.

[16] World Health Organization, 2000. *Handbook IMCI of World Health Organization*. Available at: https://iris.who.int/handle/10665/42939. Accessed November 5, 2025.

[17] Young Infants Clinical Signs Study Group, 2008. Clinical signs that predict severe illness in children under age 2 months: A multicentre study. Lancet 371: 135–142. 18191685 10.1016/S0140-6736(08)60106-3

[18] World Health Organization, 2019. *Management of the Sick Young Infant Age up to 2 Months: Facilitator Guide*. Available at: https://www.who.int/publications/i/item/who-mca-19.03. Accessed October 28, 2025.

[19] Haque KN, 2005. Definitions of bloodstream infection in the newborn. *Pediatr Crit Care Med* 6: S45–S49. 15857558 10.1097/01.PCC.0000161946.73305.0A

[20] Adediwura OA David AO Iyabode OD Olufunke BS Olusola AA, 2017. Neonatal sepsis in a Nigerian tertiary hospital: Clinical features, clinical outcome, aetiology and antibiotic susceptibility pattern. 2017. South Afr J Inf Dis 32: 127–131.

[21] Okechukwu AA Achonwa A, 2009. Morbidity and mortality patterns of admissions into the Special Care Baby Unit of University of Abuja Teaching Hospital, Gwagwalada, Nigeria. Niger J Clin Pract 12: 389–394. 20329678

[22] Garba BI Muhammad AS Mohammed BA Obasi AB Adeniji AO, 2017. A study of neonatal mortality in a specialist hospital in Gusau, Zamfara, NorthWestern Nigeria. IJTDH 28: 1–6.

[23] Camacho-Gonzalez A Spearman PW Stoll BJ, 2013. Neonatal infectious diseases: Evaluation of neonatal sepsis. Pediatr Clin North Am 60: 367–389. 23481106 10.1016/j.pcl.2012.12.003 PMC4405627

[24] Nurjadi D Eichel VM Tabatabai P Klein S Last K Mutters NT Pöschl J Zanger P Heeg K Boutin S, 2021. Surveillance for colonization, transmission, and infection with methicillin-sensitive *Staphylococcus aureus* in a neonatal intensive care unit. JAMA Netw Open 4: e2124938. 34515783 10.1001/jamanetworkopen.2021.24938 PMC8438598

[25] Reich PJ Boyle MG Hogan PG Johnson AJ Wallace MA Elward AM Warner BB Burnham C-AD Fritz SA, 2016. Emergence of community-associated methicillin-resistant *Staphylococcus aureus* strains in the neonatal intensive care unit: An infection prevention and patient safety challenge. Clin Microbiol Infect 22: 645.e1–645.e8. 10.1016/j.cmi.2016.04.013 PMC4987169 27126609

[26] Zervou FN Zacharioudakis IM Ziakas PD Mylonakis E, 2014. MRSA colonization and risk of infection in the neonatal and pediatric ICU: A meta-analysis. Pediatrics 133: e1015–e1023. 24616358 10.1542/peds.2013-3413

[27] de Man P Verhoeven BA Verbrugh HA Vos MC van den Anker JN, 2000. An antibiotic policy to prevent emergence of resistant bacilli. Lancet 355: 973–978. 10768436 10.1016/s0140-6736(00)90015-1

[28] Le J Nguyen T Okamoto M McKamy S Lieberman JM, 2008. Impact of empiric antibiotic use on development of infections caused by extended-spectrum beta-lactamase bacteria in a neonatal intensive care unit. Pediatr Infect Dis J 27: 314–318. 18316990 10.1097/INF.0b013e3181606850

[29] Cotten CM McDonald S Stoll B Goldberg RN Poole K Benjamin DK Jr., 2006. The association of third-generation cephalosporin use and invasive candidiasis in extremely low birth-weight infants. Pediatrics 118: 717–722. 16882828 10.1542/peds.2005-2677

[30] Kuppala VS Meinzen-Derr J Morrow AL Schibler KR, 2011. Prolonged initial empirical antibiotic treatment is associated with adverse outcomes in premature infants. J Pediatr 159: 720–725. 21784435 10.1016/j.jpeds.2011.05.033 PMC3193552

[31] Clark RH Bloom BT Spitzer AR Gerstmann DR, 2006. Empiric use of ampicillin and cefotaxime, compared with ampicillin and gentamicin, for neonates at risk for sepsis is associated with an increased risk of neonatal death. Pediatrics 117: 67–74. 16396862 10.1542/peds.2005-0179

[32] Cotten CM Taylor S Stoll B Goldberg RN Hansen NI Sánchez PJ Ambalavanan N Benjamin DK Jr.; NICHD Neonatal Research Network, 2009. Prolonged duration of initial empirical antibiotic treatment is associated with increased rates of necrotizing enterocolitis and death for extremely low birth weight infants. Pediatrics 123: 58–66. 19117861 10.1542/peds.2007-3423 PMC2760222

[33] Zea-Vera A Ochoa TJ, 2015. Challenges in the diagnosis and management of neonatal sepsis. J Trop Pediatr 61: 1–13. 25604489 10.1093/tropej/fmu079 PMC4375388

[34] Maina M Mwaniki P Odira E Kiko N McKnight J Schultsz C English M Tosas-Auguet O, 2020. Antibiotic use in Kenyan public hospitals: Prevalence, appropriateness, and link to guideline availability. Int J Infect Dis 99: 10–18. 32781162 10.1016/j.ijid.2020.07.084 PMC7562818

[35] Mahbuba M Joyanta KM Roman M Mahboob M Mohammad SI Samir KS, 2011. Biomarkers for diagnosis of neonatal infections: A systematic analysis of their potential as a point-of-care diagnostics. J Glob Health 1: 201–209. 23198119 PMC3484777

[36] George EN Vidya M Amita G, 2014. Biomarkers for sepsis: A review with special attention to India. BioMed Res Int 2014: 264351. 24772418 10.1155/2014/264351 PMC3977532

[37] The Lancet Microbe, 2025. Rethinking blood culture. Lancet Microbe 6: 101060. 39709976 10.1016/j.lanmic.2024.101060

[38] Mukhtar-Yola M Iliyasu Z. A review of neonatal morbidity and mortality in Aminu Kano Teaching Hospital, northern Nigeria. *Trop Doct* 37: 130–132. 17716492 10.1258/004947507781524683

[39] Dharshni P Lerusha N Khine SS-H Yesholata M, 2021. Neonatal sepsis in a tertiary unit in South Africa. *BMC Infect Dis* 21: 225. 33639864 10.1186/s12879-021-05869-3 PMC7912533

[40] Tumuhamye J Sommerfelt H Bwanga F Ndeezi G Mukunya D Napyo A Nankabirwa V Tumwine JK, 2020. Neonatal sepsis at Mulago national referral hospital in Uganda: Etiology, antimicrobial resistance, associated factors and case fatality risk. PLoS One 15: e0237085. 32776958 10.1371/journal.pone.0237085 PMC7416959

[41] Chaurasia S Sivanandan S Agarwal R Ellis S Sharland M Sankar MJ, 2019. Neonatal sepsis in South Asia: Huge burden and spiraling antimicrobial resistance. BMJ 364: k5314. 30670451 10.1136/bmj.k5314 PMC6340339

[42] Onazi SO Akeredolu FD Yakubu M Jiya MN Hano IJ, 2021. Neonatal morbidity and mortality: A 5-year analysis at Federal Medical Centre, Gusau, Zamfara State, Northwest Nigeria. ANMRP 2: 10.

[43] Abdullahi UI, 2018. Neonatal morbidity and mortality in a rural tertiary hospital in Nigeria. CHRISMED J Health Res 5: 8–10.

[44] Ogala WN Wammanda RD Zoaka HE, 1996. Analysis and outcome of admission to the special care baby unit of Ahmadu Bello University teaching hospital, Zaria. *Niger J Med* 1: 70–73.

[45] Omoigberale AI Sadoh WE Nwaneri DU, 2010. A 4 year review of neonatal outcome at the University of Benin Teaching Hospital, Benin City. Niger J Clin Pract 13: 321–325. 20857794

[46] Mustapha SS Zaidu AM Azaria NT Aliyu S Abdulkadir I, 2024. Neonatal sepsis-A peek into our findings in Northwest Nigeria: A prospective study. *Egypt Pediatric Association Gaz* 72: 53.

