# Supplementary material for: Neonatal Sepsis: Etiology, Antimicrobial Susceptibility, and Treatment Outcomes in a Tertiary Hospital in Jos, Nigeria
Source: Am J Trop Med Hyg. 2025 Nov 20;114(2):207–15. doi: 10.4269/ajtmh.24-0127 (PMC12874867; doi:10.4269/ajtmh.24-0127)
Supplement: Supplemental Materials [file tpmd240127.SD1.pdf]

NNS PROFORMA

NNS STUDY, JUTH

S/N0: JUTH/NNS/000….

SECTION A (BIODATA, OBS ANTHROPOMETRY)

1. Hospital N0: ……………..
2. Age (Hours/ days) at admin
3. Sex (a) Male (b) Female (c) Ambiguous
4. Gestational age (weeks)
5. Birth weight (kg)
6. Place of birth (a) In-born (b) Out-born
7. Mode of delivery (a) SVD (b) CS (c) Assisted
8. Birth attendant (a) (Midwife) (b) Doctor (c) CHEW (d) None
9. OFC (cm)
10. Length (cm)
11. PROM
12. Vaccination at status
13. Peripartum fever
14. Chorioamnionitis
15. Cord care
16. Temperature at birth (^{O}C)
17. Admitting RBS
18. PCV
19. TSB (g/dl)
20. HBV status
21. HCV status
22. HIV status
23. Blood culture test (a) POS (b) NEG (c) Not done (d) Not requested for (e) Bottle stock-out
24. Pathogen isolated
25. Cord swabs
26. Eye swabs

27. Skin swabs

28. Urine culture

29. CSF culture

30. Admitting diagnosis

31. Final diagnosis

32. Sepsis severity (a) Yes (b) No

33. Antimicrobial sensitivity

34. Antimicrobial resistance

35. Antimicrobials administered

36. Duration of hospital stay

37. Postmortem done (a) Yes (b) No (c) Not requested for

38. Postmortem cause of death

SECTION B (MATERNAL & FAMILY CHARCTERISTICS)

39. Maternal age (years)

40. Highest educational attainment

41. Occupation

42. Parity

43. Educational status

44. ANC attendance

45. HBV status

46. HCV status

47. HIV status

48. Blood group

49. Fathers age (years)

50. Highest education attainment

51. Occupation

SECTION C

52. Treatment outcome (a) Dead (b) Alive (c) Morbidity (a) Yes (b) No. if yes, specify

THANK YOU
